# Supplementary material for: Dissecting the enhancer gene regulatory network in early Drosophila spermatogenesis
Source: Nat Commun. 2025 Jul 23;16:6766. doi: 10.1038/s41467-025-62046-9 (PMC12284225; doi:10.1038/s41467-025-62046-9)
Supplement: Supplementary file 1 — Supplementary Information [file 41467_2025_62046_MOESM1_ESM.pdf]

Dissecting the enhancer gene regulatory network in early *Drosophila* spermatogenesis

SUPPLEMENTARY INFORMATION

Patrick van Nierop y Sanchez[1], Pallavi Santhi Sekhar[1], Kerem Yildirim[1], Tim Lange[1], Laura Zoe Kreplin[1], Vigneshwarr Muruga Boopathy[1], Stephanie Rosswag de Souza[1], Kim Dammer[1], David Ibberson[2], Qian Wang[1], Katrin Domsch[1], Anniek Stokkermans[3], Shubhanshu Pandey[1], Petra Kaspar[1], Rafael Martinez-Gallegos[1], Xuefan Gao[1], Aakriti Singh[1], Natalja Engel[4], Fillip Port[5], Michael Boutros[5], Josephine Bageritz[5], Ingrid Lohmann[1#]

AFFILIATIONS

[1]Heidelberg University, Centre for Organismal Studies (COS) Heidelberg, Department of Developmental Biology and Cell Networks - Cluster of Excellence, Heidelberg, Germany.

[2]Deep Sequencing Core Facility, BioQuant, Heidelberg University, Heidelberg, Germany.

[3]Hubrecht Institute-KNAW, Utrecht, Netherlands.

[4]Heidelberg University, Centre for Organismal Studies (COS) Heidelberg, Department of Stem Cell Niche Heterogeneity, Heidelberg, Germany.

[5]German Cancer Research Center (DKFZ), Div. Signaling and Functional Genomics and University of Heidelberg, Dept. of Cell and Molecular Biology, Heidelberg, Germany.

[#] corresponding author: ingrid.lohmann@cos.uni-heidelberg.de

Supplementary Figures

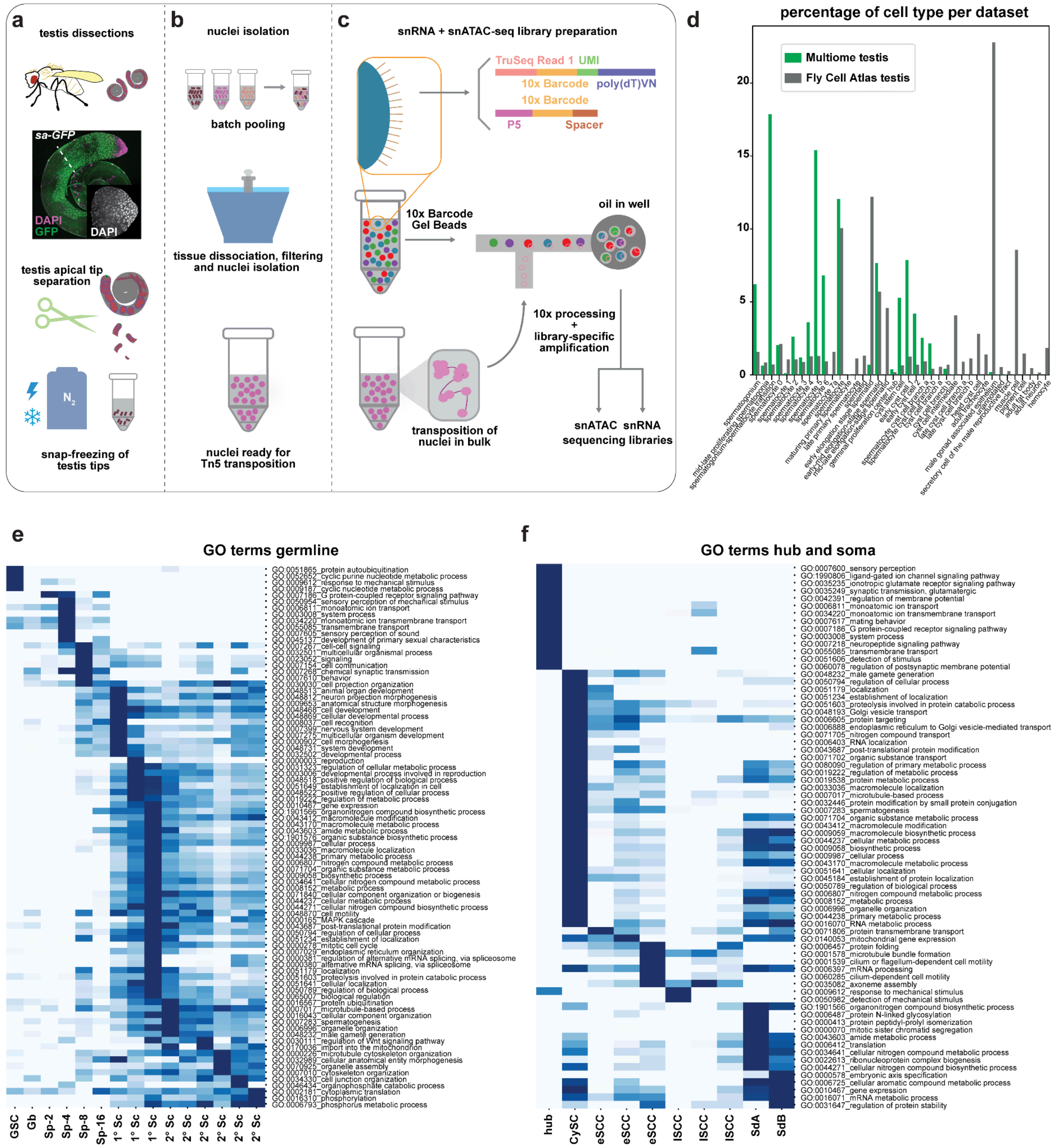

Supplementary Figure 1: Workflow for enrichment of apical testis tip nuclei for multiomic analysis.

(a) Testes of 1 day old *w*^{1118} flies were dissected and apical tips were cut. Inset depicts the approximate location of the cuts with nuclei marked by DAPI (magenta) and spermatocytes by *sa-GFP* (green). Zoom-in shows an exemplary DAPI-stained cut testis-tip. The cut testis-tips are collected and rapidly snap-frozen by liquid nitrogen. **(b)** Sample batches are pooled and the tissue is dissociated mechanically

through douncing, creating a nuclei suspension. **(c)** Pooled samples were mechanically dissociated by douncing to generate a nuclei suspension. **(c)** Nuclei were transposed with Tn5 and co-encapsulated with 10x barcoded beads via microfluidics. After RNA and ATAC-specific amplification, libraries were sequenced. **(d)** Barplot showing enrichment of early testis cell types in the current dataset (green) compared to the Fly Cell Atlas (gray). **(e)** Heatplot depicting the scaled enrichment of GO terms per germline cell stage (white to blue: low to high enrichment). **(f)** Equivalent analysis for hub cells and somatic cell stages.

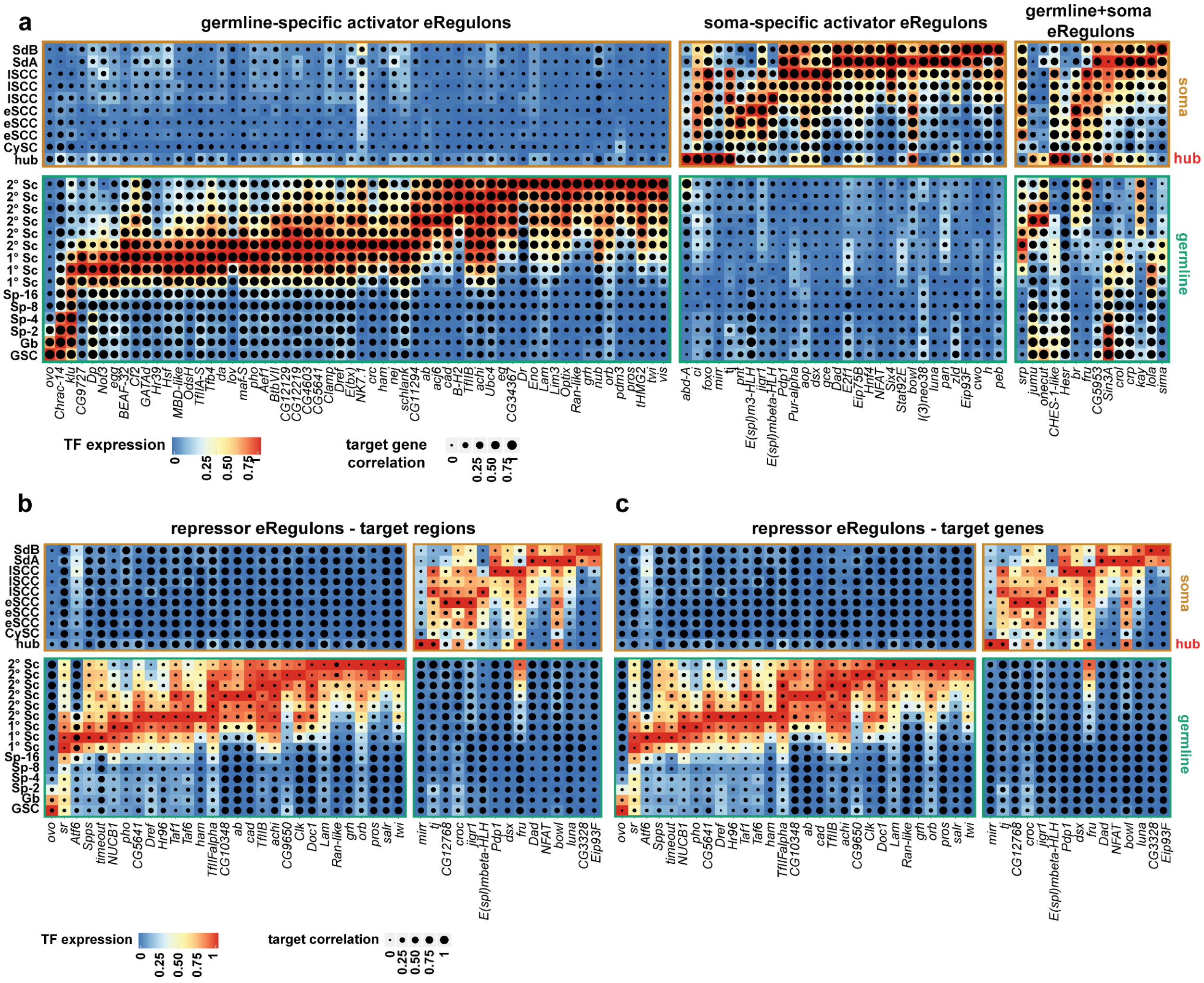

Supplementary Figure 2: Activator and repressor eRegulons define lineage trajectories.

(a) Dotplot heatmap showing expression levels of activating eRegulon TFs (color) and correlation with target gene expression (dot size), ordered by peak TF expression. **(b, c)** Dotplot heatmaps of repressor eRegulons, displaying TF expression (color) and correlation with accessibility of target regions (a) or gene expression (b). eRegulons are ordered by peak TF expression, revealing lineage-restricted activity.

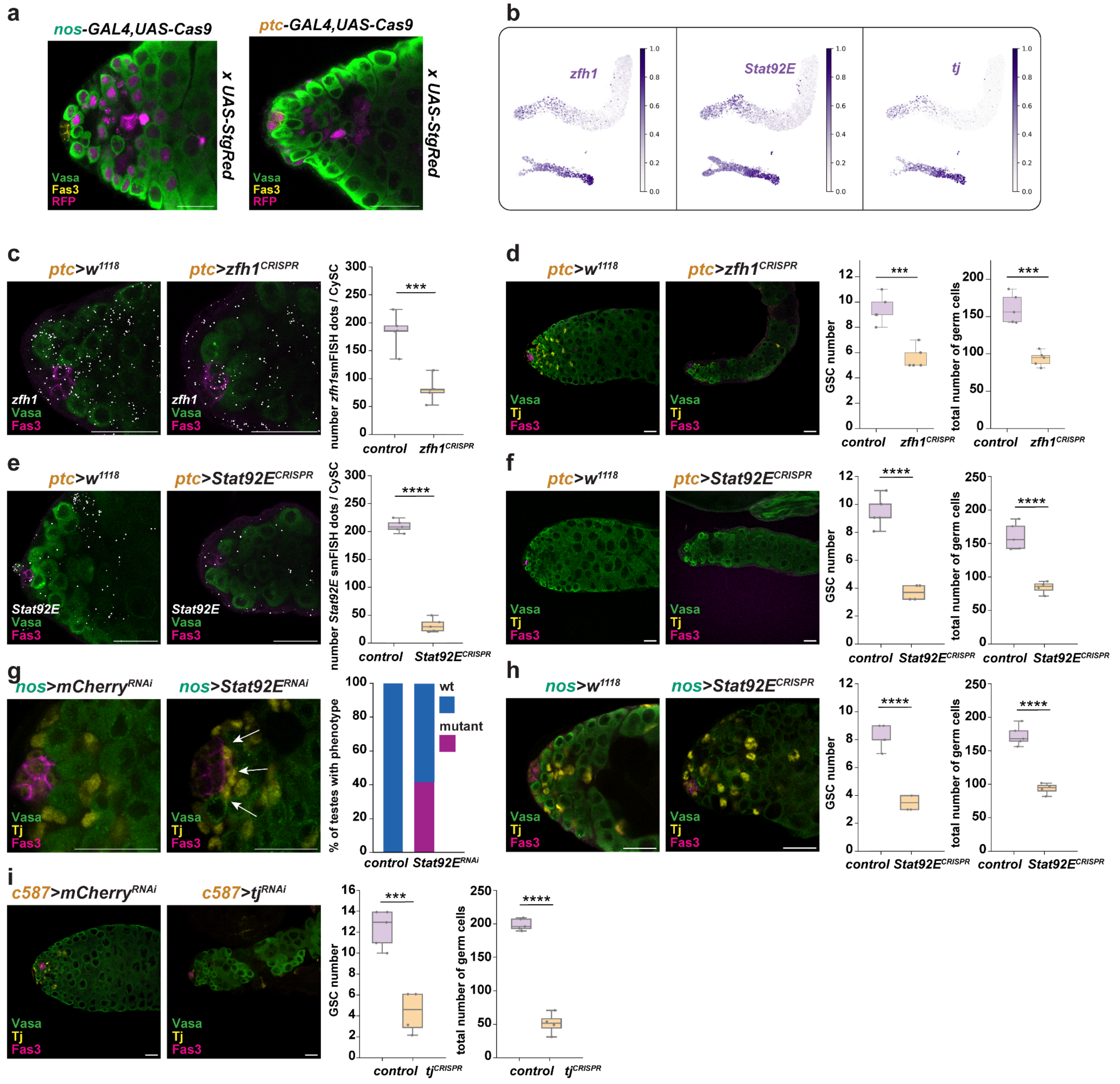

Supplementary Figure 3: TF perturbations *in vivo* result in phenotypic testes.

(a) Representative images of the apical tip of testes where the *UAS-StingerRed* (*UAS-StgRed*) transgene is driven by the *nos-GAL4,UAS-Cas9* (left) or *ptc-GAL4,UAS-Cas9* (right) drivers, showing that the transgene is expressed exclusively in the germline (left) or the somatic (right) lineage. **(b)** Expression of indicated genes projected onto the UMAP; expression levels are shown as increasing intensity of purple. **(c)** Left: Representative smFISH images of *zfh1* transcripts in control and soma-specific *zfh1* knockout (KO) testes. Right: Quantification of *zfh1* smFISH signal across genotypes. **(d)** Left: Representative images of control and soma-specific *zfh1* KO testes. Right: Quantification of GSC number, GSC diameter, and total germ cell number. **(e)** Left: Representative smFISH images of *Stat92E* in control and soma-specific *Stat92E* KO testes. Right: Quantification of *Stat92E* smFISH signal. **(f)** Left: Control and *crc*

KO testes. Right: Quantification of GSC number, GSC diameter, and total germ cell number. **(g)** Left: Representative images of *Stat92E* in control and germline-specific *Stat92E* RNAi testes. Arrows highlight the row of accumulated Tj positive cells between hub cells and GSCs. Right: Quantification of the occurrence of the phenotype shown on the left side. **(h)** Left: Representative images of control and germline-specific *Stat92E* KO testes. Right: Quantification of GSC number, GSC diameter, and total germ cell number. **(i)** Left: Representative smFISH images of *klu* in control and germline-specific *klu* KO testes. Right: Quantification of *klu* smFISH signal. **(j)** Left: Representative images of control and soma-specific *tj* RNAi testes. Right: Quantification of GSC number, GSC diameter, and total germ cell number. In all images, Vasa (green) labels the germline, Fas3 (magenta) marks hub cells, Tj (yellow) labels the somatic lineage and smFISH signals are shown in white. Scale bars, 20 µm. (c: n=5, 5; d: n=5, 5; e: n=5, 5; f: n=5, 4; g: n=10, 10; h: n=5, 5; i: n=5, 4) Boxplots represent median values within the Q1-Q3 range, while whiskers include the 0-100 percentiles. P-values are calculated with two-sided independent t-tests. (*P < 0.05; **P < 0.01; ***P < 0.001; ****P < 0.0001). Exact p-values can be found in the Source data file. See also Supplementary Tables 1-2, Supplementary Data 4. Source data is provided as a Source Data file (Figure S3).

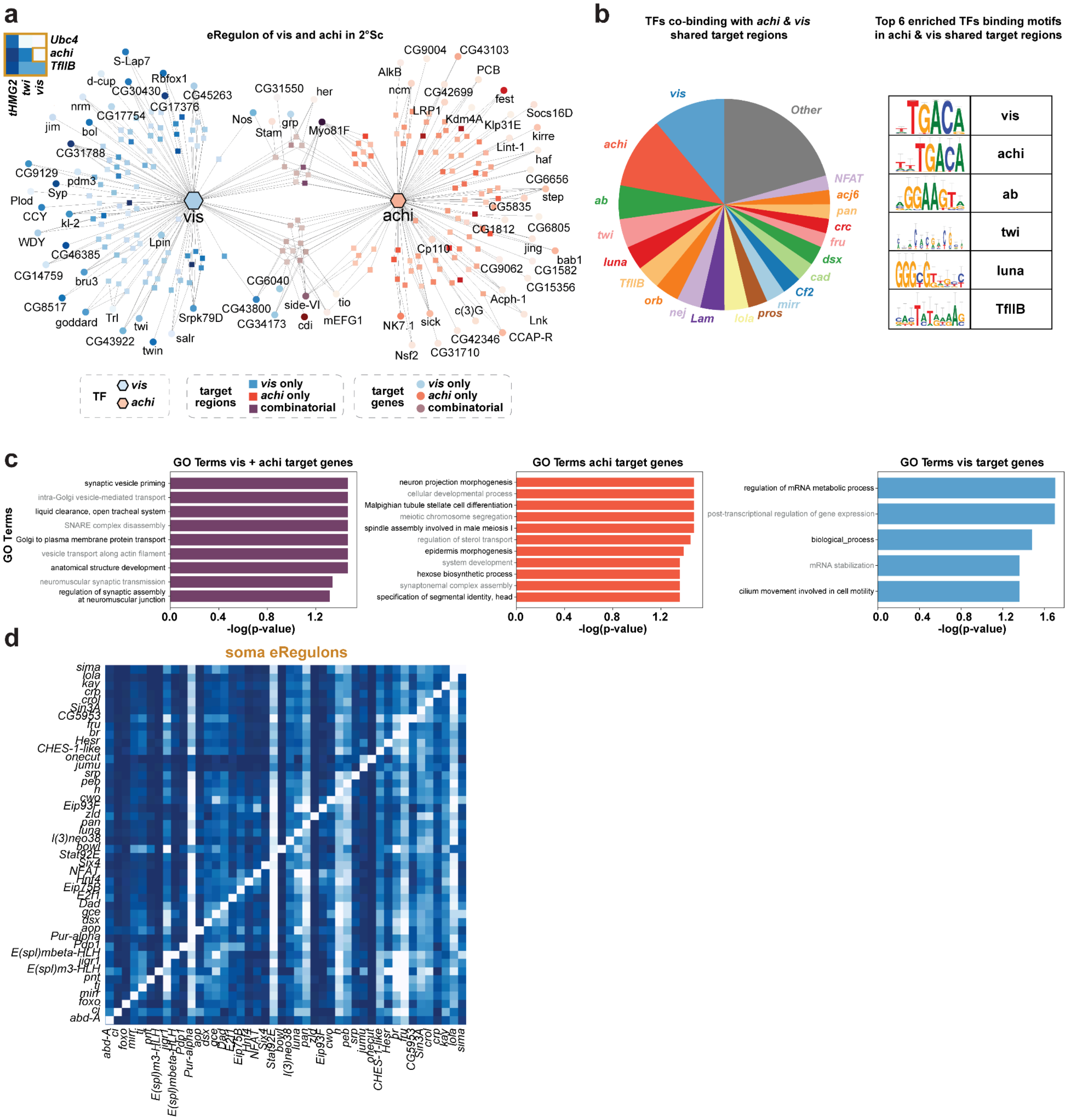

Supplementary Figure 4: Analysis of the *vis* and *achi* networks reveal joint target regulation.

(a) Visualization of the *vis* and *achi* eRegulons in 2°Sc with TFs, regions and genes colored according to their mode of inferred regulation. The top 100 linkages based on TF-to-gene importance for each TF have been plotted. Three distinct classes of genes are separable, exclusively regulated by Vis (blue), Achi (red) and combinatorially regulated by both factors (purple). **(b)** Pie chart showing TF motif content in Vis–Achi co-regulated regions; less frequent TFs are grouped as "Other". Table lists the top six enriched motifs. **(c)** GO term enrichment for three classes of target genes reveals distinct regulatory programs and shared

functions. **(d)** Heatmap of normalized intersection scores among soma eRegulons based on shared regulatory regions.

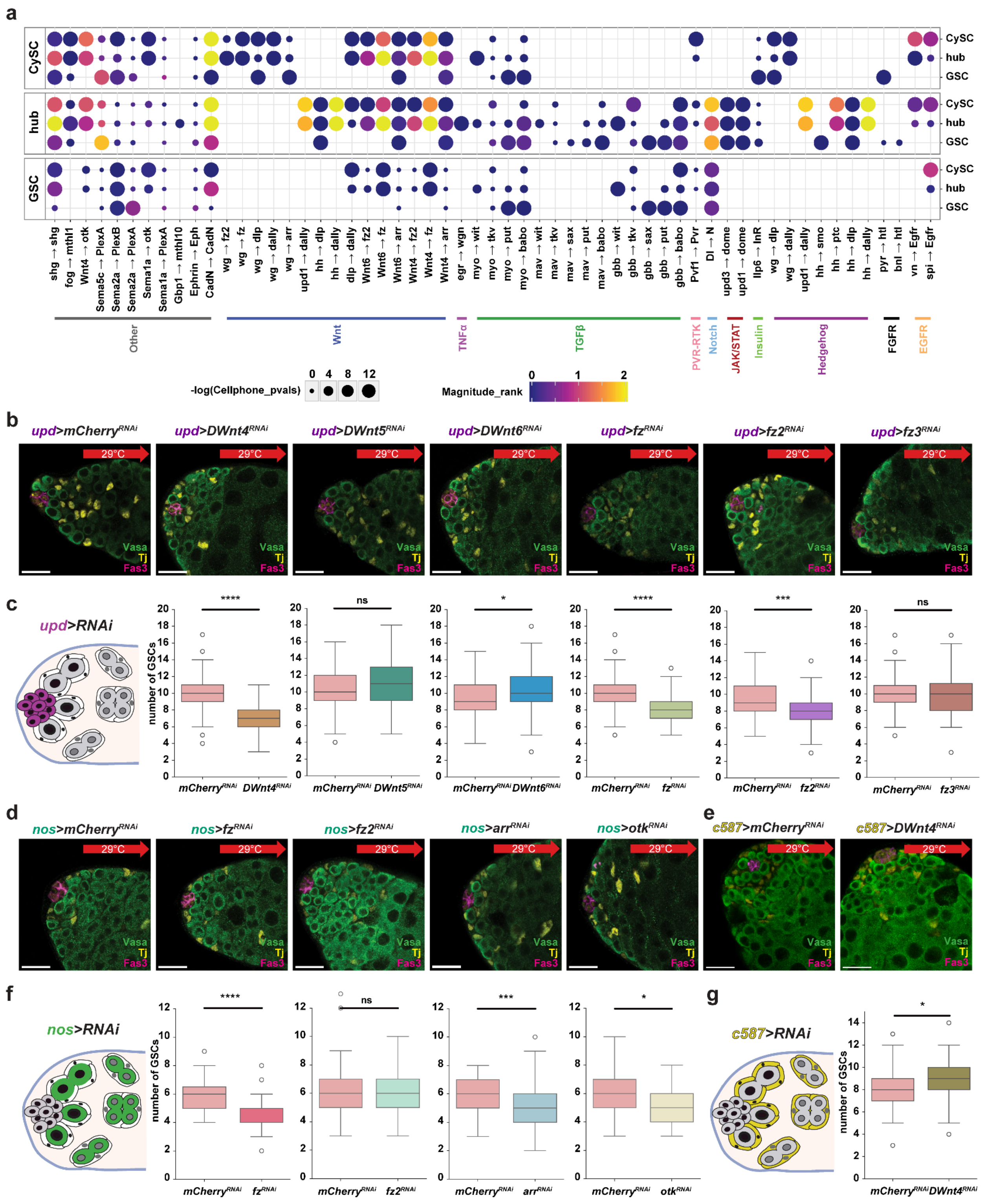

Supplementary Figure 5: Characterization of the Wnt pathway in the *Drosophila* testis.

(a) Extended dotplot of predicted ligand-receptor interactions at the stem cell niche. Dot size corresponds with the -log(Cellphone p-value) metric, color scale corresponds with the LIANA+ magnitude rank score, values above 2 are displayed as the maximum. **(b)** Representative images of testes after continuous

interference with Wnt pathway genes (crosses kept at 29°C from embryogenesis onwards till phenotypic analysis in 7-days old adult testes) in hub cells (*upd-GAL4>RNAi*) are shown. **(c)** Quantification of GSC numbers in age-matched control and knockdown testes in indicated genotypes. **(d, e)** Representative images of testes after continuous interference with Wnt pathway genes in early gemline cells (*nos-GAL4>RNAi*) (d) or somatic cells (*c587-GAL4>RNAi*) (e) are shown. **(f, g)** Quantification of GSC numbers in age-matched control and knockdown testes in indicated genotypes. The germline is marked by Vasa (green), early soma by Tj (yellow), and hub cells by Fas3 (magenta). Scale bars, 20 µm. (c, f, g: n≥66). Boxplots represent median values within the Q1-Q3 range, while whiskers include 1.5*IQR ranges. P-values are calculated with two-sided Mann-Withney tests. (*P < 0.05; **P < 0.01; ***P < 0.001; ****P < 0.0001). Exact p-values can be found in the Source Data file. See also Supplementary Tables 1-2. Source data is provided as a Source Data file (Figure S5).

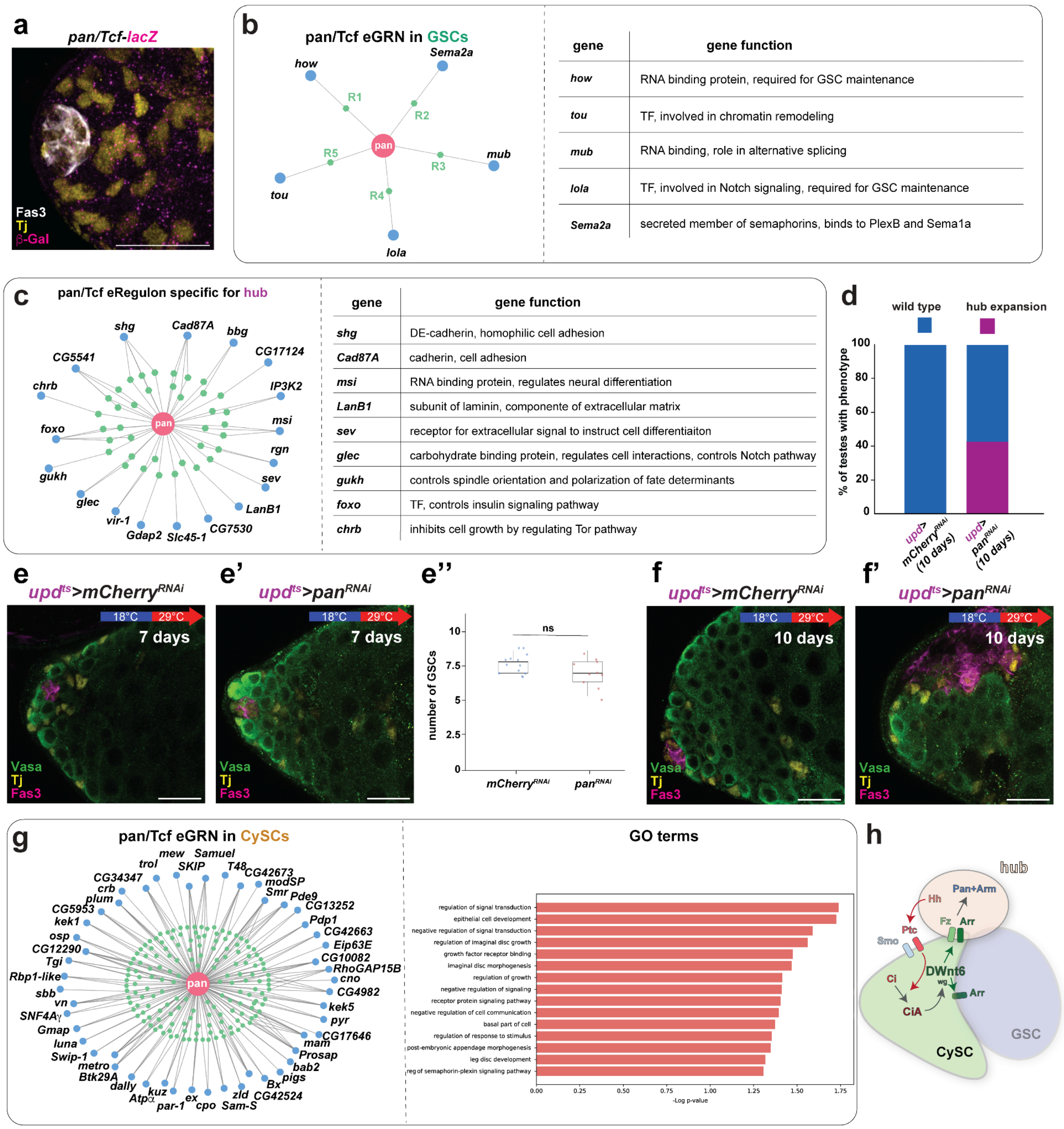

Supplementary Figure 6: Pan/Tcf regulates early spermatogenesis via distinct regulatory networks.

(a) Expression of a Wnt reporter containing multiple Tcf binding sites driving *lacZ*; hub cells (Fas3, white), somatic cells (Tj, yellow), and reporter activity (β-Gal, red) are shown. **(b, c)** Left: Visualization of Pan/Tcf eRegulons in GSCs (b) and hub cells (c); right: GO terms associated with target genes in each lineage. **(d)** Quantification of phenotypes following hub-specific, adult-only *pan* knockdown 10 days after eclosion. **(e, e')** Representative images of adult-specific, hub-targeted *pan* knockdown by RNAi, 7 days post-eclosion. **(e'')** Quantification of GSC numbers in the genotypes shown in (e, e'). **(f, f')** Representative

images of adult-specific, hub-targeted *pan* knockdown by RNAi, 10 days post-eclosion. **(g)** Left: Visualization of Pan/Tcf eRegulons in CySCs; right: GO terms associated with target genes in this lineage. **(h)** Model of interdependency between hub and CySC lineages via Hedgehog and Wnt signaling. The germline is highlighted by Vasa (green), the early somatic lineage by Tj (yellow), hub cells are highlighted by Fas3 (red). Scale bars, 20 µm. (d: n=10, 10; e'': n= 15, 10). Boxplots represent median values within the Q1-Q3 range, while whiskers include 1.5*IQR ranges. P-values are calculated with two-sided independent t-tests. (*P < 0.05; **P < 0.01; ***P < 0.001; ****P < 0.0001). See also Supplementary Tables 1-2. Source data is provided as a Source Data file (Figure S6).

Supplementary Tables

Supplementary Table 1 - *Drosophila melanogaster* stocks used in this study.

Type	current_symbol	sgRNA_1	sgRNA_2	Provider
U6:3-gRNA	Stat92E	CACCATGTACCCGGTAACCA	ATGTGTGAGCTCCGGCTCTT	Fillip Port
U6:3-gRNA	crc	GCTGTACTGGGACCTCAAGA	TACTTTGTTTGCGGGTACTA	Fillip Port
U6:3-gRNA	ovo	AAAGGTGCCAATATCGATCT	GCTCCCGTTGGCGGTGAATT	Fillip Port
U6:3-gRNA	Mad	TGTGTTCGATTCCACATCGT	TGGCGAGGAACGAGTACTGG	Fillip Port
U6:3-gRNA	zfh1	CTGGGTACACTTGACCAGGA	GGGCATGCTCTGCTGAATGA	Fillip Port
U6:3-gRNA	klu	GCCCCTGCCCCATCCCCCGCC GG	TGATGGGTCCGTGCCTGATGAG G	Fillip Port
**Type**	**current_symbol**	**Description**	**external ID**	**Provider**
UAS-RNAi	tj	y[1] sc[*] v[1] sev[21]; P{y[+t7.7] v[+t1.8]=TRiP.HMS01069}attP2	34595	Bloomington
UAS-RNAi	Stat92E	y[1] v[1]; P{y[+t7.7] v[+t1.8]=TRiP.JF01266}attP2	31318	Bloomington
UAS-RNAi	mcherry	y[1] sc[*] v[1] sev[21]; P{y[+t7.7] v[+t1.8]=VALIUM20-mCherry}attP2	35785	Bloomington
UAS-RNAi	Wnt4	P{KK102348}VIE-260B	104671	VDRC
UAS-RNAi	Wnt5	y[1] v[1]; P{y[+t7.7] v[+t1.8]=TRiP.HM05020}attP2	28534	Bloomington
UAS-RNAi	Wnt6	y[1] sc[*] v[1] sev[21]; P{y[+t7.7] v[+t1.8]=TRiP.HM05236}attP2	30493	Bloomington
UAS-RNAi	fz	w[1118]; P{GD4614}v43077	43077	VDRC
UAS-RNAi	fz2	y[1] sc[*] v[1] sev[21]; P{y[+t7.7] v[+t1.8]=TRiP.HMS05675}attP40	67863	Bloomington
UAS-RNAi	fz3	y[1] sc[*] v[1] sev[21]; P{y[+t7.7] v[+t1.8]=TRiP.GLC01626}attP2	44468	Bloomington
UAS-RNAi	arr	y[1] v[1]; P{y[+t7.7] v[+t1.8]=TRiP.JF01260}attP2	31313	Bloomington

UAS-RNAi	otk	y[1] sc[*] v[1] sev[21]; P{y[+t7.7] v[+t1.8]=TRiP.HMC04139}attP2	55869	Bloomington
UAS-RNAi	pan	*y[1] v[1]; P{y[+t7.7] v[+t1.8]=TRiP.HMS02015}attP40/CyO*	40848	Bloomington
UAS-RNAi	ci	P{KK100760}VIE-260B	105620	VDRC
GAL4-Driver	upd-Gal4	P{w[+mW.hs]=GawB}E132, w[*]	26796	Bloomington
GAL4-Driver	nos-Gal4	w[1118]; P{w[+mC]=GAL4::VP16-nos.UTR}CG6325[MVD1]	4937	Bloomington
GAL4-Driver	C587-Gal4	P{w[+mW.hs]=GawB}C587, w[*]	67747	Bloomington
GAL4-Driver	nos-Gal4;vasaEGFP	TI{TI}vasKI.EGFP ; P{w[+mC]=GAL4::VP16-nos.UTR}CG6325[MVD1]	base 4937	Kerem Yildirim
GAL4-Driver	C587-Gal4;vasaEGFP	c587-Gal4 ; TI{TI}vasKI.EGFP/(CyO-WeeP)	base 67747	Kerem Yildirim
GAL4-Driver, UAS-Cas9	; UAS-Cas9; hh-Gal4	P{ry[+t7.2]=hsFLP}12, y[1] w[*]; P{y[+t7.7] w[+mC]=UAS-uMCas9}attP40; P{Gal4}hh-Gal4/TM6B	340019	Fillip Port
GAL4-Driver, UAS-Cas9	; ptc-Gal4, UAS-Cas9	P{ry[+t7.2]=hsFLP}12, y[1] w[*]; P{Gal4}ptc-Gal4 P{y[+t7.7] w[+mC]=UAS-uMCas9}attP40	340022	Fillip Port
GAL4-Driver, UAS-Cas9	; UAS-Cas9; nos-Gal4	w[*]; P{y[+t7.7] w[+mC]=UAS-uMCas9}attP40, P{GAL4::VP16-nos.UTR}CG6325 MVD1/TM6B	340010	Fillip Port
GAL4-Driver, tubulin Gal80ts	Upd-Gal4ts	*upd-Gal4 ; tubGal80ts/CyO*	base 26796	Pallavi
GAL4-Driver, tubulin Gal80ts	c587-Gal4ts	*c587-Gal4 ; tubGal80ts/ CyO*	base 67747	Pallavi
GFP_line	vasaEGFP.KI	TI{TI}vas[EGFP.KI]	118616	Kyoto DGGR
control	w1118	w[1118]	3605	Bloomington
UAS-stgRed	UAS-stringer	w[*]; P{w[+mC]=UAS-RedStinger}6, P{w[+mC]=UAS-FLP.Exel}3, P{w[+mC]=Ubi-p63E(FRT.STOP)St	28281	Bloomington

		inger}15F2		
TCF-reporter	tcf-lacz	w[*];<br>P{w[+mC]=6TCF-Helper-lacZ}2	68166	Bloomington

Supplementary Table 2 - Antibodies used in this study.

Antigen	Host	Provider	dilution	external link
Vasa	rabbit	Santa Cruz	1:200	https://www.scbt.com/p/vasa-antibody-d-260
Fas3	mouse	DSHB	1:100	https://dshb.biology.uiowa.edu/7G10-anti-Fasciclin-III
Tj	guinea pig	Dorothea Godt	1:10000	https://doi.org/10.1242/dev.089896
Org-1	rat	Manfred Frasch	1:100	https://doi.org/10.1242/dev.073890
GFP	chicken	Rockland	1:300	https://www.rockland.com/categories/primary-antibodies/gfp-antibody-600-901-215/
Ecad	mouse	DSHB	1:300	https://dshb.biology.uiowa.edu/5D3
β-Galactosidase	rabbit	Kappel labs	1:1000	https://www.bioz.com/result/rabbit%20anti%20%CE%B2%20galactosidase/product/Cappel%20Laboratories
eya	mouse	DHSB	1:100	https://dshb.biology.uiowa.edu/eya10H6_4

Supplementary Methods

SINGLE NUCLEI ISOLATION PROTOCOL

Preparation of buffers:

0. *For sequencing add RNAse inhibitor, otherwise replace with RNAse free water.*
1. Dissolve Digitonin at 65°C.
2. Put douncers in ice, pestles on white tissue paper on ice.
3. Prepare all buffers according to 10x protocol (CG000366 – Rev D) (next page)
4. Pre-coat three flat bottom – conical 2 ml tubes with 1ml Wash Buffer, you can reuse the buffer between tubes, keep on ice. Then proceed to coat the douncer(s) with the same buffer from the tubes. *This is all to prevent the nuclei from sticking to the tube and douncers too much*.
5. Add 400 µl cold Lysis Buffer and immediately homogenize using a Pellet Pestle.
6. Transfer the homogenate into a cold douncer and incubate for 5-10 minutes on ice.
7. Pipette mix 10x with a 1ml pre-coated pipette tip in Wash Buffer.
8. Incubate another 5-10 minutes on ice.
9. Add 500 µl cold Wash Buffer to the lysed cells. *This slows down further lysis.*
10. Dounce 25x with the tight pestle directly. *Optimize for sample.*
11. Pass the suspension through a pre-coated 20µm cell strainer, into a pre-coated 2ml tube. *Do not forget to precoat the pipette tip.*
12. Pass the suspension though a 40µm Flowmi Cell Strainer (BAH136800040) into another pre-coated 2ml tube. *Again, pre-coat the pipette tip before aspiration.*
13. Centrifuge in a swing bucket centrifuge at 700g for 7 minutes at 4°C. *Optimize for cell type by reducing force while keeping centrifugation time at a minimum. You can check whether all nuclei of interest pelleted down by checking the supernatant for nuclei.*
14. Remove supernatant and add 0.9ml Wash Buffer and gently resuspend the cell pellet with a pre-coated pipette tip.
15. Repeat steps 11 and 12 two more times.
16. Pass the suspension through one last (Flowmi) filter, into a third pre-coated 2ml tube.
17. Centrifuge one last time, and resuspend in Nuclei Buffer, according to the amount of material you have.
18. Count the nuclei, preferably with a fluorescent marker, according to your needs.

Diluted Nuclei Buffer<br>Maintain at 4°C	Stock	Final	1 ml
Nuclei Buffer* (20X)	20X	1X	50 µl
DTT	1000 mM	1mM	1 µl
RNase inhibitor (check vendor-specific stock concentration)	40 U/µl	1 U/µl	25 µl
Nuclease-free Water	-	-	924 µl
**Wash Buffer**<br>**Prepare fresh, maintain at 4°C**	**Stock**	**Final**	**4 ml**
Tris-HCl (pH 7.4)	1 M	10 mM	40 µl
NaCl	5 M	10 mM	8 µl
$MgCl_2$	1 M	3 mM	12 µl
BSA	10%	1%	400 µl
Tween-20	10%	0.1%	40 µl
DTT	1000 mM	1 mM	4 µl
RNase inhibitor	40 U/µl	1 U/µl	100 µl
Nuclease-free Water	-	-	3.40 ml
**Lysis Buffer**<br>**Prepare fresh, maintain at 4°C**	**Stock**	**Final**	**2 ml**
Tris-HCl (pH 7.4)	1 M	10 mM	20 µl
NaCl	5 M	10 mM	4 µl
$MgCl_2$	1 M	3 mM	6 µl
Tween-20	10%	0.1%	20 µl
Nonidet P40 Substitute (if using Sigma (74385) 100% solution, prepare a 10% stock)	10%	0.1%	20 µl
Digitonin (incubate at 65°C to dissolve precipitate before use)	5%	0.01%	4 µl
BSA	10%	1%	200 µl
DTT	1000 mM	1 mM	2 µl
RNase inhibitor 40 U/µl	40 U/µl	1 U/µl	50 µl
Nuclease-free Water	-	-	1.67 ml

From 10x Genomics protocol (CG000338 Revision E).

Suggested Fluorescent markers for nuclei detection:

**Compound**	**Emission max.**	**Dilution**	**Note**
DAPI	470	1:500	Stains debris well
SYTOX Green	504	1:1000	Low background, good for widefield microscopy
SYTOX Orange	570	1:1000	Low background, good for widefield microscopy
DRAQ7	697	1:1000	Very low background
