# Supplementary material for: Dissecting the enhancer gene regulatory network in early Drosophila spermatogenesis
Source: Nat Commun. 2025 Jul 23;16:6766. doi: 10.1038/s41467-025-62046-9 (PMC12284225; doi:10.1038/s41467-025-62046-9)
Supplement: Supplementary file 2 — Description Of Additional Supplementary File [file 41467_2025_62046_MOESM2_ESM.pdf]

Description of Additional supplementary files

Supplementary Data 1 - All barcodes of the testis multiome that pass the thresholds as specified in the Methods.

Supplementary Data 2 - Multiome 10x barcodes manually excluded as specified in the Methods.

Supplementary Data 3 - Multiome 10x barcodes included for the construction of the testis eGRN.

Supplementary Data 4 - smFISH oligos used in this study
